# Serum MicroRNAs Reflect Injury Severity in a Large Animal Model of Thoracic Spinal Cord Injury

**DOI:** 10.1038/s41598-017-01299-x

**Published:** 2017-05-03

**Authors:** Seth Tigchelaar, Femke Streijger, Sunita Sinha, Stephane Flibotte, Neda Manouchehri, Kitty So, Katelyn Shortt, Elena Okon, Michael A. Rizzuto, Ivana Malenica, Amanda Courtright-Lim, Andrew Eisen, Kendall Van Keuren-Jensen, Corey Nislow, Brian K. Kwon

**Affiliations:** 1 International Collaboration on Repair Discoveries (ICORD), University of British Columbia, Vancouver, British Columbia Canada; 2 Faculty of Pharmaceutical Sciences, University of British Columbia, Vancouver, British Columbia Canada; 3 Translational Genomics, Phoenix, Arizona United States of America; 4 Acorda Therapeutics, Ardsley, New York United States of America; 5 Vancouver Spine Surgery Institute, Department of Orthopedics, University of British Columbia, Vancouver, British Columbia Canada

## Abstract

Therapeutic development for spinal cord injury is hindered by the difficulty in conducting clinical trials, which to date have relied solely on functional outcome measures for patient enrollment, stratification, and evaluation. Biological biomarkers that accurately classify injury severity and predict neurologic outcome would represent a paradigm shift in the way spinal cord injury clinical trials could be conducted. MicroRNAs have emerged as attractive biomarker candidates due to their stability in biological fluids, their phylogenetic similarities, and their tissue specificity. Here we characterized a porcine model of spinal cord injury using a combined behavioural, histological, and molecular approach. We performed next-generation sequencing on microRNAs in serum samples collected before injury and then at 1, 3, and 5 days post injury. We identified 58, 21, 9, and 7 altered miRNA after severe, moderate, and mild spinal cord injury, and SHAM surgery, respectively. These data were combined with behavioural and histological analysis. Overall miRNA expression at 1 and 3 days post injury strongly correlates with outcome measures at 12 weeks post injury. The data presented here indicate that serum miRNAs are promising candidates as biomarkers for the evaluation of injury severity for spinal cord injury or other forms of traumatic, acute, neurologic injury.

## Introduction

Spinal cord injury (SCI) is a devastating condition, often resulting in life-long disability. The patient population with SCI comprises an extremely heterogeneous group with considerable differences in injury mechanism, location, severity, and patient genetics. Each year, over 17,000 individuals in North America and many thousands more around the world suffer an acute traumatic SCI and are left paralyzed^1^. There are nearly 290,000 individuals living with SCI in the United States alone. The estimated lifetime costs for one SCI ranges from $1–4 million, depending on the severity of injury. A small number of acute SCI clinical trials have been conducted; yet none have shown convincing efficacy^2–5^. As a consequence, neuro-restorative treatment options are limited for acute SCI patients.

A fundamental limitation of such clinical trials is their singular reliance on standardized measures of neurologic function for patient enrollment, stratification, and treatment assessment. The baseline clinical assessment of SCI and measurement of neurologic impairment can be inaccurate or impossible to conduct due to intoxication, sedation, or concomitant injuries. In a review of over 400 acute SCI patients, we previously showed that concomitant injuries and co-morbidities would have excluded at least 30% of patients from participation into an acute clinical trial^6^. The inability to determine a baseline functional severity of neurologic impairment further limits the pool of “recruitable” patients for acute SCI clinical trials of new therapies, and is a major impediment to the translation of such therapies. In those who can be reliably examined within the first few hours post-injury and the baseline level of neurological impairment ascertained, the variability in spontaneous neurologic recovery is high, forcing investigators to enroll large numbers of patients to have sufficient statistical power^7^. Accordingly, new treatments that show promise in experimental models of SCI are extremely difficult to validate in acute SCI patients.

Biological markers of spinal cord injury that objectively stratify the severity of cord damage would greatly facilitate clinical trials of novel therapies for acute SCI. Additionally, such biomarkers may be able to predict spontaneous neurologic recovery over time with greater precision, sensitivity and reproducibility than the standard clinical examination, which in turn would reduce the number of patients needed to sufficiently power clinical trials. Biological outcome measures that can be applied between porcine models and human patients could accelerate the preclinical development and subsequent clinical evaluation of novel therapeutics. It is recognized within and outside the SCI field that biomarkers that enhance patient enrolment and provide surrogate outcome measures would increase the pace of clinical trials and improve the chances of successfully demonstrating efficacy, thus moving us more quickly towards effective treatments. For example, it has been estimated that the use of biomarkers in the selection of subjects for early Alzheimer’s trials could reduce sample size by 67% and trials costs by 60% as compared to trials depending solely upon clinical measures^8^. For these reasons, there has been considerable research interest in establishing cerebrospinal fluid (CSF) and serum biomarkers after acute SCI, traumatic brain injury, and other acute and chronic neurologic conditions^9–17^. For example, Kwon *et al*.^9^ have shown significant differences between the proteins IL-6, tau, S100β, and GFAP within the CSF of patients with acute SCI. While these proteins (and their levels in SCI) show promise in defining the severity of SCI, the procedure of acquiring CSF through a lumbar puncture is relatively invasive. A blood-borne biological marker that could characterize injury severity and predict outcome would therefore be desirable.

In this work, we investigated the potential for miRNA (short, ~22 nucleotide non-coding RNAs that post-transcriptionally regulate gene expression) measured in serum and CSF to serve as biomarkers of injury severity after acute traumatic SCI in a pig (*Sus scrofa)* model. MiRNA have emerged as attractive biomarker candidates due to their stability in fluids, phylogenetic similarities, and tissue specificity^17–19^. Using this large animal model of contusion plus compression SCI, we collected CSF and serum samples from 16 animals over 5 days and performed next-generation sequencing on the miRNA population within these biological fluids to determine if changes in miRNA can be used as biomarkers of injury severity. We found a severity-dependent increase in the number and magnitude of miRNA within the serum of pigs, that was most pronounced at 1 and 3 days post injury (dpi). The total amount of miRNA at these time points significantly correlated with injury severity, measured by force of impact, as well as with functional outcome scores at 12 weeks post injury (wpi), measured using the Porcine Thoracic Injury Behaviour Score (PTIBS).

## Results

### Study Overview

**Figure 1:**
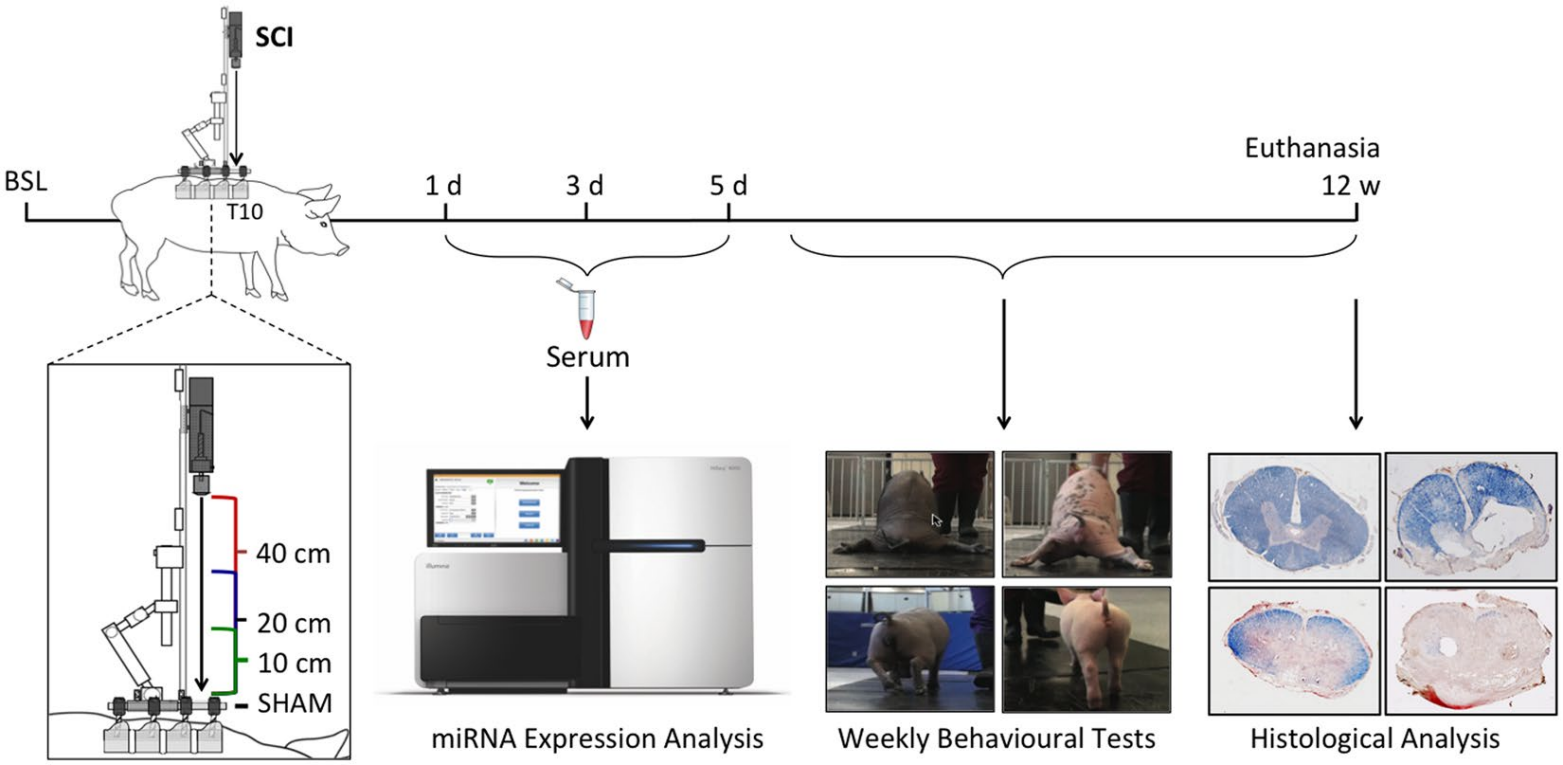
Study Outline. Miniature Yucatan pigs received either SHAM laminectomy (black), or a contusion spinal cord injury using a weight dropped from 40 (red), 20 (blue), or 10 cm (green), followed by 5 minutes of compression. For miRNA expression profiling, CSF and serum samples were collected before injury (baseline (BSL)) and at 1, 3, and 5 days post injury (dpi). Functional recovery was assessed weekly for 12 weeks using Porcine Thoracic Injury Behaviour Scale (PTIBS)^12^. At 12 weeks post-injury, animals were euthanized and the spinal cord processed for spared white and gray matter quantification.

Female miniature Yucatan pigs (n = 12) received a contusion spinal cord injury by dropping a 50 g weight from a height of either 10 cm (mild), 20 cm (moderate), or 40 cm (severe) onto the exposed spinal cord, followed by 5 minutes of compression with an additional 100 g weight (150 g total) (Summarized in Fig. 1). An additional group of pigs (n = 4) received all aspects of the surgical procedure (including laminectomy) but without any spinal cord injury, thereby serving as “SHAM” or “NON-SCI” controls. Impact forces recorded with a force sensor were consistent within injury height groups, and correlated with injury height (Table 1). Samples of cerebrospinal fluid (CSF) and serum were collected at baseline (BSL), 15 minutes prior to injury, and then at 1, 3, and 5 days post injury (dpi) (Table 1). Total RNA was isolated and microRNA (miRNA) libraries were created for expression profiling using next-generation sequencing. Animals performed weekly functional recovery tests for 12 weeks following spinal cord injury (SCI). At 12 weeks post injury, animals were euthanized and spinal cords were collected for histological analysis of spared tissue.

**Table 1:** Study parameters.

Drop Height (cm)	n	Impact Force (N)	Sample Type	Number of Samples (n)			
				BSL	1 dpi	dpi	5 dpi
10	4	19.22 ± 1.70	Serum	4	4	4	4
			CSF	4	4	4	4
20	4	30.04 ± 2.52	Serum	4	4	4	4
			CSF	4	4	4	4
40	4	47.87 ± 6.93	Serum	4	4	4	4
			CSF	4	4	4	4
NA (SHAM)	4	NA	Serum	4	4	4	4
			CSF	4	4	4	4

The number of animals in each injury group is shown with their respective injury heights and recorded impact forces in Newtons (N), along with the number of samples collected for each sample type (serum or CSF) and time point (baseline (BSL), days post-injury (dpi)).

### Behavioural outcomes

**Figure 2:**
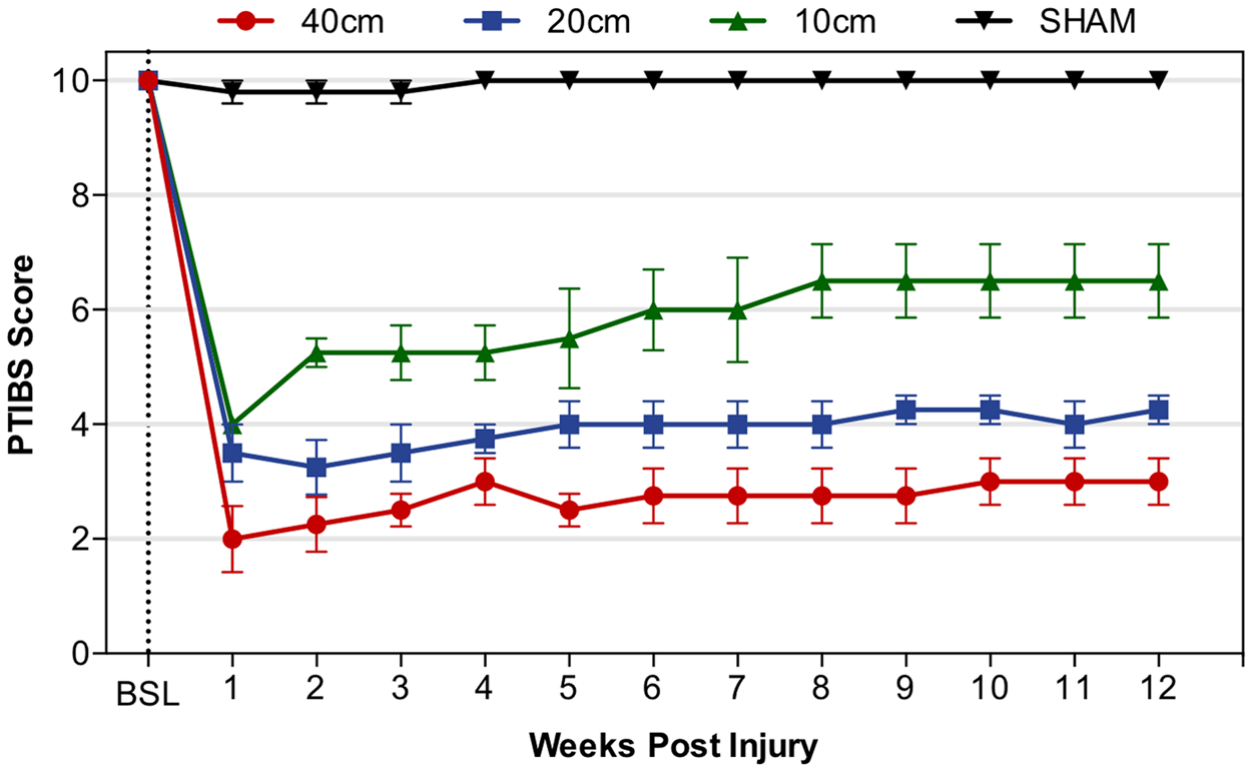
Effect of injury severity on locomotor recovery after spinal cord injury. Porcine Thoracic Injury Behaviour Scale (PTIBS) scores were measured before injury (baseline (BSL)) and weekly for 12 weeks post injury for the 40 (red), 20 (blue), 10 cm (green), and SHAM (black) groups. Datapoints represent mean ± SEM for n = 4 animals per group (see Table 1).

To measure the recovery of gross locomotor performance, hindlimb function was assessed using the Porcine Thoracic Injury Behaviour Scale (PTIBS) designed by Lee *et al*.^20^. Scoring was performed pre-operatively and then on a weekly basis post-injury (Fig. 2). Prior to injury, all animals achieved a baseline score of 10, indicative of normal hindlimb function and locomotion. Following SCI, locomotor function was most severely impaired at 1 week post-injury, with mean PTIBS scores of 2, 3.2, and 4 for the severe, moderate, and mild SCI groups respectively, and 10 for the Sham group (Fig. 2). As expected, lowest PTIBS scores were observed at the greatest injury severity. At all post-injury time points (from week 1 to 12) all injured animals were substantially and significantly impaired compared to baseline values, independent of their injury severity, while the SHAM group had little to no impairment. By 12 wpi, the mean PTIBS scores were 3, 4.25, 6.5, and 10 for the severe, moderate, mild SCI and SHAM groups, respectively. Injury height had a significant effect on PTIBS scores F(3, 169) = 803.9, p < 0.0001 with significant differences between all groups from week 6 to 12. This data provides evidence to confirm the generation of four distinct injury-severity groups that correlated with behavioral recovery.

### Histological Outcomes

**Figure 3:**
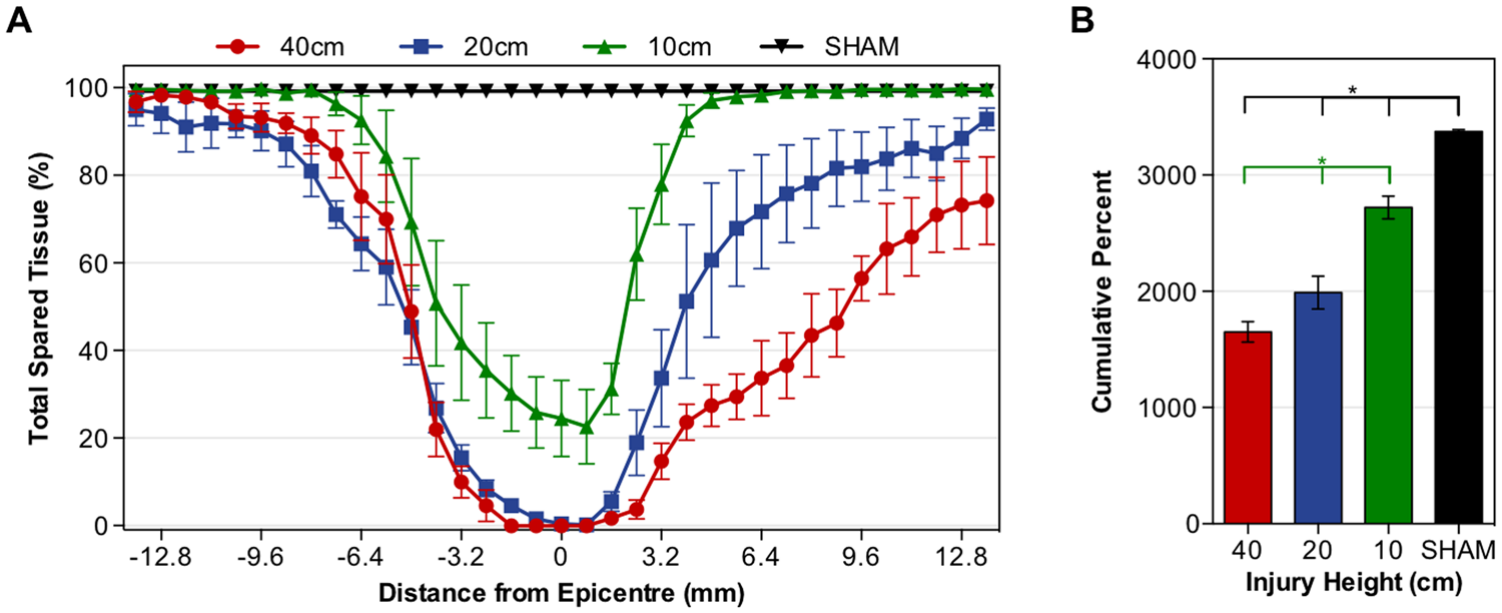
Effect of injury severity on tissue sparing 12 weeks after spinal cord injury. Spared tissue was measured using Eriochrome Cyanine stained sections from 13.6 mm rostral and 13.6 mm caudal to the site of injury. (**A**) The total spared tissue in percent, relative to the entire area of the spinal cord of the same section was calculated for the 40 (red), 20 (blue), 10 cm (green), and SHAM (black) groups. (**B**) The total amount of tissue sparing (cumulative percent) was calculated using the area under the curve of total spared tissue (%) for the 40 (red), 20 (blue), 10 cm (green), and SHAM (black) groups. Asterisks indicate sparing for which significant differences were found between groups with ANOVA. Data is presented as means ± SEM for n = 4 animals per group. SCI, spinal cord injury.

To assess the amount of spared (healthy) tissue within the spinal cord, spared gray matter and white matter were quantified by manually tracing the respective cross-sections of spinal cord, 0.8 mm apart, as previously described by Lee *et al*.^20^. Figure 3A shows the total percent spared tissue. Total percent spared tissue was calculated by taking the sum of the spared white and gray matter and dividing that value by the total area of the spinal cord on its respective cross-section. The height of injury had a significant effect on the amount of spared tissue (greater height, less spared tissue), calculated as the area under the curve of the total percent spared tissue over the region from 13.6 mm rostral to 13.6 mm caudal to the site of injury F(3, 12) = 63.46, p < 0.0001. Cumulative spared tissue was greatest in the SHAM group (3,380 ± 16.0 cumulative%) compared to the mild (2,720 ± 98.0 cumulative%), moderate (1,990 ± 140 cumulative%) and severe (1,650 ± 88.0 cumulative%) SCI groups, respectively (p = 0.0022, p < 0.0001, p < 0.0001) (Fig. 3B). The cumulative spared tissue was significantly greater in the mild SCI group as compared to the moderate SCI group (p = 0.0008) and the severe SCI group (p < 0.0001). While the spared tissue was significantly higher between the 3.2 to 11.2 mm sections caudal to the epicenter in the moderate SCI group compared to the severe SCI group, there were no statistical differences between the cumulative areas (p = 0.12). This data provides evidence that the four *behaviorally* distinct groups are also reflected by four distinct injury-severity groups with regards to histologically measured tissue damage.

### MicroRNA detection in CSF and serum

**Figure 4:**
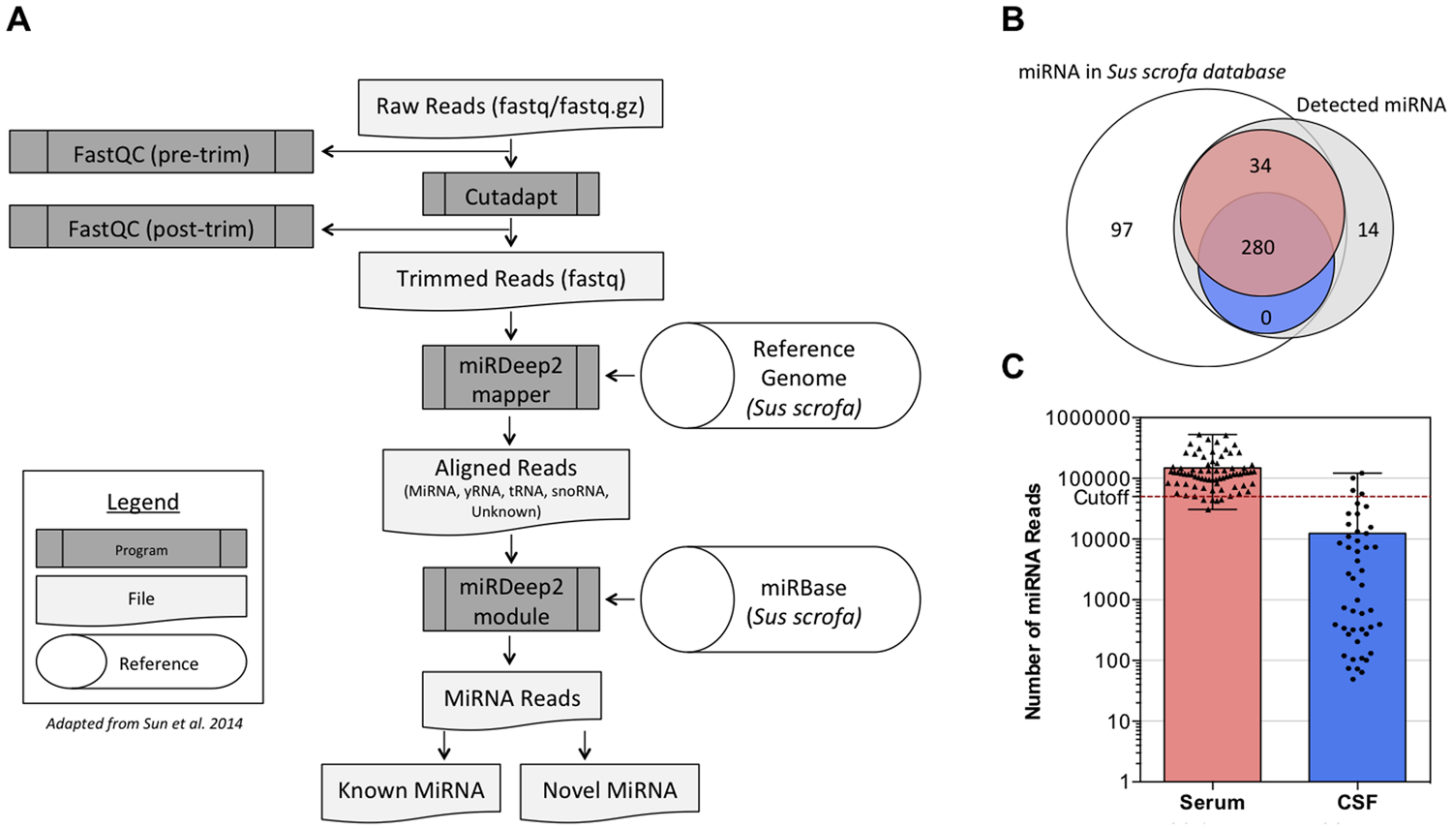
(**A**) Bioinformatic Pipeline. Raw reads were assessed for quality using FastQC before and after adapter trimming with the Cutadapt package. Trimmed reads were mapped to the pig (*Sus scrofa*) reference genome using the miRDeep2 mapper. Aligned reads were then mapped to the miRBase miRNA database using the miRDeep2 module. MiRDeep2 gives a matrix list of known and novel miRNA. (**B**) Detected miRNA. 314 out of the total 411 known *Sus scrofa* miRNA (white) were identified across all serum (red, 314 miRNA) and CSF (blue, 280 miRNA) samples, while 14 novel miRNA (grey) that do not yet exist in the *Sus scrofa* miRNA database were identified. (**C**) Number of miRNA reads for all samples. 60 serum samples (red) had over the cutoff of 50,000 miRNA reads, only 4 CSF (blue) samples had above 50,000 reads, and 11 CSF samples had over 10,000 miRNA reads.

MiRNA libraries were created from total RNA isolated from CSF or serum. RNA concentrations in CSF are reported to be between 15–30 ng per mL, while serum has 10–60 ng per mL^21^. These relatively low levels of total RNA required the protocol modifications described by Burgos *et al*.^21^ for Illumina’s TruSeq Small RNA Library Preparation, which requires 1 ug of total input RNA. This enabled us to generate sequencing libraries for 64 CSF and 64 serum samples. Libraries were sequenced to generate short single-end reads, which were processed using the Mayo Clinic’s Comprehensive Analysis Pipeline for miRNA Sequencing Data (CAP-miRSeq) bioinformatic pipeline (Fig. 4A).

Trimmed reads were first aligned to the pig S*us scrofa* (Version 10.2) reference genome^22^. The resulting aligned reads were then mapped to the miRNA database (Version 21), miRBase. The *Sus scrofa* miRNA database from miRBase contains 411 known, mature miRNAs. Most of these (305 miRNA) are also found in the human miRNA database, which contains over 1,800 mature miRNA. Aligning our sequencing reads to the human database did not significantly increase the number of detected mature miRNA, so all further analyses used the *Sus scrofa* database.

Without using any read-count cutoff, we detected a total of 314 previously identified miRNA that were expressed in one or more time points across all serum samples (Fig. 4B), which represents over 75% of all known pig miRNA. Interestingly, there were no miRNA uniquely detected in CSF. 280 miRNA were detected in CSF, all of which were also found in serum, while there were an additional 34 miRNA uniquely detected in serum. Using the confidence cutoffs proposed by Burgos *et al*.^23^ that require a novel miRNA to be present in at least 30% of samples, our analysis also detected 14 putative novel miRNA in serum (Fig. 4B, Supplementary Table 1).

For profiling changes in miRNA, we only considered samples with a total miRNA read count per sample above 50,000, and an average read count for any individual miRNA of over 5 counts. As shown in Supplementary Figure 1, for samples below this cutoff the Spearman correlation drops below 0.9. 60 serum samples were above this cutoff, unfortunately, only four CSF samples had over 50,000 miRNA reads (Fig. 4C). Overall, sequence coverage was much lower for CSF samples; the majority of samples had less than 10,000 miRNA reads. This read count was not improved by additional sequencing. The two likely explanations for this result is 1) The abundance of miRNA in CSF is very low^21^, or 2) there may be inhibitors of PCR in the pig CSF samples that interfere with library preparations^24^. While miRNA profiling was not successful in the majority of CSF samples, a correlation analysis was performed between the normalized CSF miRNA counts and serum miRNA counts of all detected genes (Supplementary Figure 2). The profile of CSF miRNA significantly correlated with the profile of miRNA within serum (r = 0.9620, p < 0.0001). In addition, because we found a large overlap between the miRNA content of serum and CSF samples, we proceeded to focus exclusively on serum samples for our further temporal analyses.

### Identification of Serum MicroRNAs Related to SCI

**Figure 5:**
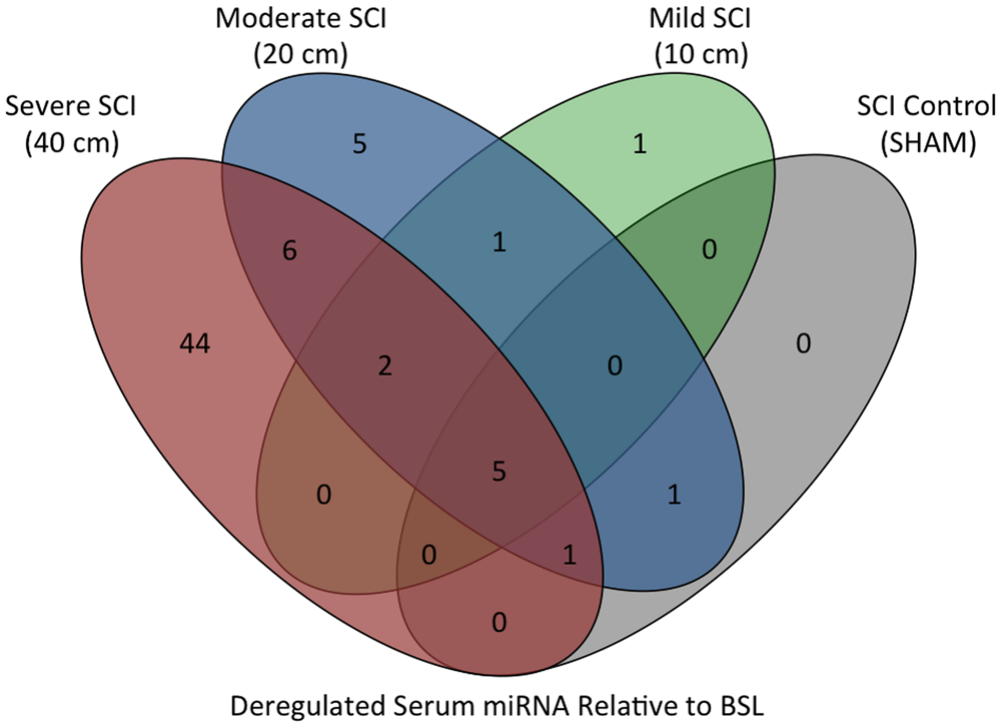
Venn diagram showing numbers of deregulated serum miRNA after severe (40 cm, red), moderate (20 cm, blue), mild (10 cm, green) injury, or SHAM surgery (Non-CNS Injury control, black). Deregulated miRNA were determined by multiple t-tests, while adjusting the p-value using the Benjamini-Hochberg method.

To determine if miRNA levels were significantly altered following SCI in a severity-dependent fashion, the log_2_ fold-change for serum miRNAs was determined, relative to pre-injury baseline levels. MiRNAs with greater than a log_2_ fold change of one, (representing a 2-fold change), and adjusted p-value < 0.05 were identified (Supplementary Table 2). From this analysis, we identified a total of 58 significantly elevated miRNA in the severe SCI group, 21 in the moderate SCI group, 9 in the mild SCI group, and 7 miRNA in the SHAM group. In addition, there was one miRNA that was significantly decreased in the moderate and mild SCI groups. The numbers of significantly elevated miRNA that were common or unique to each group are shown in a Venn diagram in Fig. 5.

**Figure 6:**
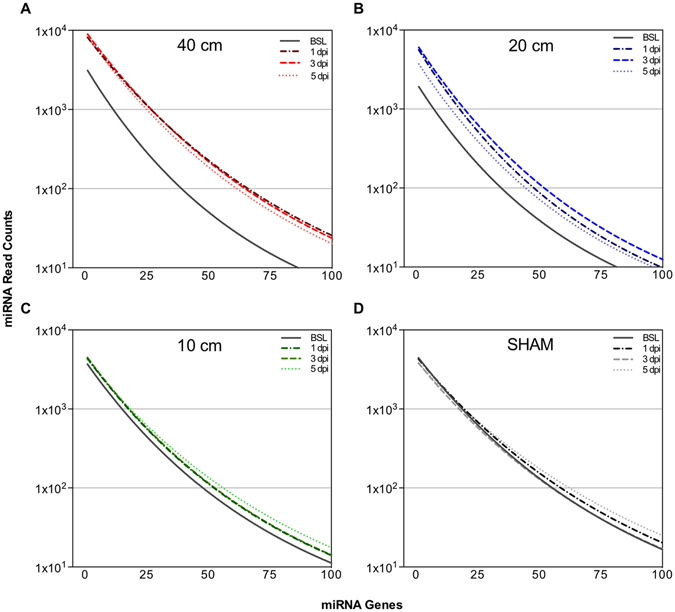
Effect of Injury Severity on Global miRNA Expression. Smoothed lines showing the trend in global miRNA expression at BSL (solid lines), and at 1, 3, and 5 dpi (dashed lines) in each of the (**A**) 40 cm, (**B**) 20 cm, (**C**) 10 cm, and (**D**) SHAM groups. Datapoints represent the smoothed read counts for the top 100 miRNA genes for n = 4 animals per group and time point (see Table 1).

Since all but one significantly altered miRNA increased after injury, we investigated the global change in miRNA across the top 100 most abundant miRNA. The aim of this was to determine if, while possibly not significant, all of the expressed miRNA had increased after injury, or just those that were found to be significant. The top 100 was used as a cutoff due to the fact that below this, miRNA had fewer than 10 counts. Figure 6 shows the profile of the 100 most abundant miRNA, generated from the average normalized read counts (Supplementary Figure 3). Interestingly, in the severe and moderate SCI groups, we found that miRNA levels across all 100 genes were elevated in response to injury, compared with BSL (Fig. 6A,B). In contrast, no increase was seen in the global miRNA levels in the mild SCI or SHAM groups (Fig. 6C,D).

**Figure 7:**
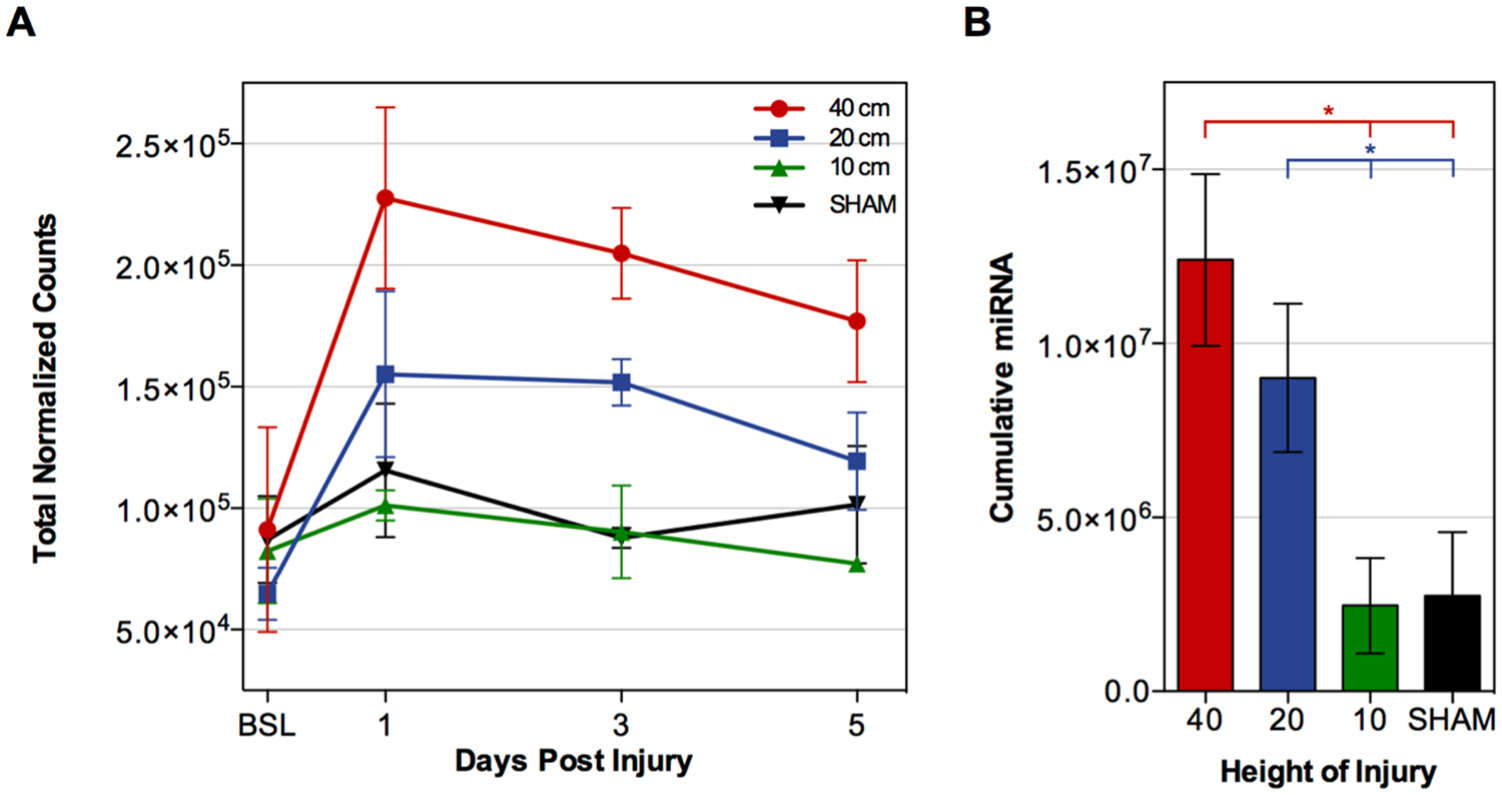
Effect of Injury Severity on total Systemic miRNA. (**A**) The total number of normalized miRNA counts in the 40 cm (red), 20 cm (blue), 10 cm (green), and SHAM (black) groups. Data points represent the average of the normalized total miRNA reads for n = 4 animals per group and time point (see Table 1). (**B**) The cumulative miRNA expression over time in the 40 cm (red), 20 cm (blue), 10 cm (green), and SHAM (black) groups. Asterisks indicate significant differences determined by ANOVA. Data is presented as means ± SEM.

We explored if the total number of normalized miRNA reads, quantified as a measure of injury severity in each group (Fig. 7A), could serve as a potential measure of severity. Injury severity had a significant effect on serum miRNA levels as measured by ANOVA F(3, 12) = 6.25, p = 0.0084. In the severe SCI group, the total amount of miRNA was significantly elevated compared to BSL (9.12 × 10^4^ ± 4.21 × 10^4^) at 1 (2.28 × 10^5^ ± 3.74 × 10^4^, p = 0.0004), 3 (2.05 × 10^5^ ± 1.86 × 10^4^, p = 0.0031), and 5 dpi (1.77 × 10^5^ ± 2.51 × 10^4^, p = 0.0341). The moderate SCI group had significantly elevated levels of miRNA compared to BSL (6.49 × 10^4^ ± 1.08 × 10^4^) at 1 (1.55 × 10^5^ ± 3.42 × 10^4^, p = 0.024) and 3 dpi (1.52 × 10^5^ ± 2.01 × 10^4^, p = 0.0307). In contrast, the mild SCI and SHAM groups did not have significantly different levels of miRNA at any time points after injury (p > 0.999 for all). Between groups, the total miRNA was significantly higher in the severe SCI group (2.28 × 10^5^ ± 3.74 × 10^4^) compared to the mild SCI (1.01 × 10^4^ ± 6.27 × 10^3^, p = 0.0024) and SHAM (1.16 × 10^5^ ± 2.75 × 10^4^, p = 0.0071) groups at 1 dpi. At 3 dpi, the severe SCI (2.05 × 10^5^ ± 1.86 × 10^4^) group had significantly higher total miRNA than the mild SCI (9.03 × 10^4^ ± 1.92 × 10^4^, p = 0.0057) and SHAM (8.79 × 10^4^ ± 4.23 × 10^3^, p = 0.0056) groups. At 5 dpi, the severe SCI group (1.77 × 10^5^ ± 2.51 × 10^4^) had significantly higher total miRNA than the mild SCI group (7.73 × 10^4^ ± 3.11 × 10^3^, p = 0.024).

Due to the stability of serum miRNAs, we predicted that the systemic miRNA load would accumulate over-time, and this was confirmed by quantifying the cumulative levels of miRNA over the 5 days post injury (Fig. 7B). Cumulative miRNA in the severe SCI group (1.24 × 10^7^ ± 2.47 × 10^6^) was significantly higher than the mild SCI (2.76 × 10^6^ ± 1.37 × 10^6^, p = 0.0074) and SHAM groups (2.90 × 10^6^ ± 1.83 × 10^6^, p = 0.0081) but not the moderate group (9.01 × 10^6^ ± 1.83 × 10^6^, p = 0.056). MiRNA levels in the moderate SCI group (9.01 × 10^6^ ± 2.14 × 10^6^) were significantly higher than the mild SCI and SHAM groups (p = 0.039, p = 0.046, respectively).

### Serum MicroRNAs at 1 and 3 dpi Correlate with Functional Outcome Measures of Injury Severity

In order to determine which time points would most accurately identify injury severity, we investigated the degree to which total miRNA levels at each time point correlate with injury severity outcome measures. Correlations between total miRNA levels, PTIBS scores at 12 wpi, and histological outcome measurements were made. Additionally, the comparison between the correlations of impact force and histological outcome or PTIBS, using the Pearson correlation coefficients, was made. Histological outcome (as measured by total percent spared tissue) and PTIBS scores at 12 wpi, were compared to BSL, 1, 3, and 5 dpi total miRNA levels.

Expectedly, the amount of miRNA at BSL, before injury, did not correlate with PTIBS scores (r = 0.043 p = 0.88 respectively) (Supplementary Figure 4). In contrast, following SCI, total miRNA at 1 dpi significantly correlated with PTIBS scores (r = −0.56 p = 0.023) (Supplementary Figure 4), while the total miRNA at 3 dpi correlated strongly with PTIBS scores (r = −0.79 p = 0.0002) (Supplementary Figure 4). The total miRNA did not reach a significant level of correlation with PTIBS scores at 5 dpi (r = −0.46, p = 0.075) (Supplementary Figure 4).

Prior to injury, miRNA levels at BSL did not correlate with total percent spared tissue (r = −0.031 p = 0.91 respectively) (Supplementary Figure 5). Following SCI, total miRNA at 1 dpi significantly correlated with total percent spared tissue (r = −0.62 p = 0.011) (Supplementary Figure 5), and at 3 dpi, total miRNA correlated well with total percent spared tissue (r = −0.79 p = 0.0003) (Supplementary Figure 5). Finally, at 5 dpi, the total level of miRNA significantly correlated with total percent spared tissue (r = −0.58, p = 0.018) (Supplementary Figure 5).

These results suggest that total miRNA at 1 and 3 dpi is directly related to injury severity and has strong predictive value regarding the cellular integrity of the spinal cord at the site of injury as well as functional recovery observed at 12 wpi. The correlation of miRNA with outcome measures was reduced by 5 dpi with regards to both behavioural outcome and histological outcome suggesting that miRNA are most predictive of outcome early after injury. As expected, the force of injury significantly correlated with PTIBS scores at 12 wpi, and histological outcome (r = −0.93 p < 0.0001, r = −0.96 p < 0.0001, respectively) (Supplementary Figure 6).

### Accuracy of miRNAs in Distinguishing Injury Severity after Traumatic SCI

**Figure 8:**
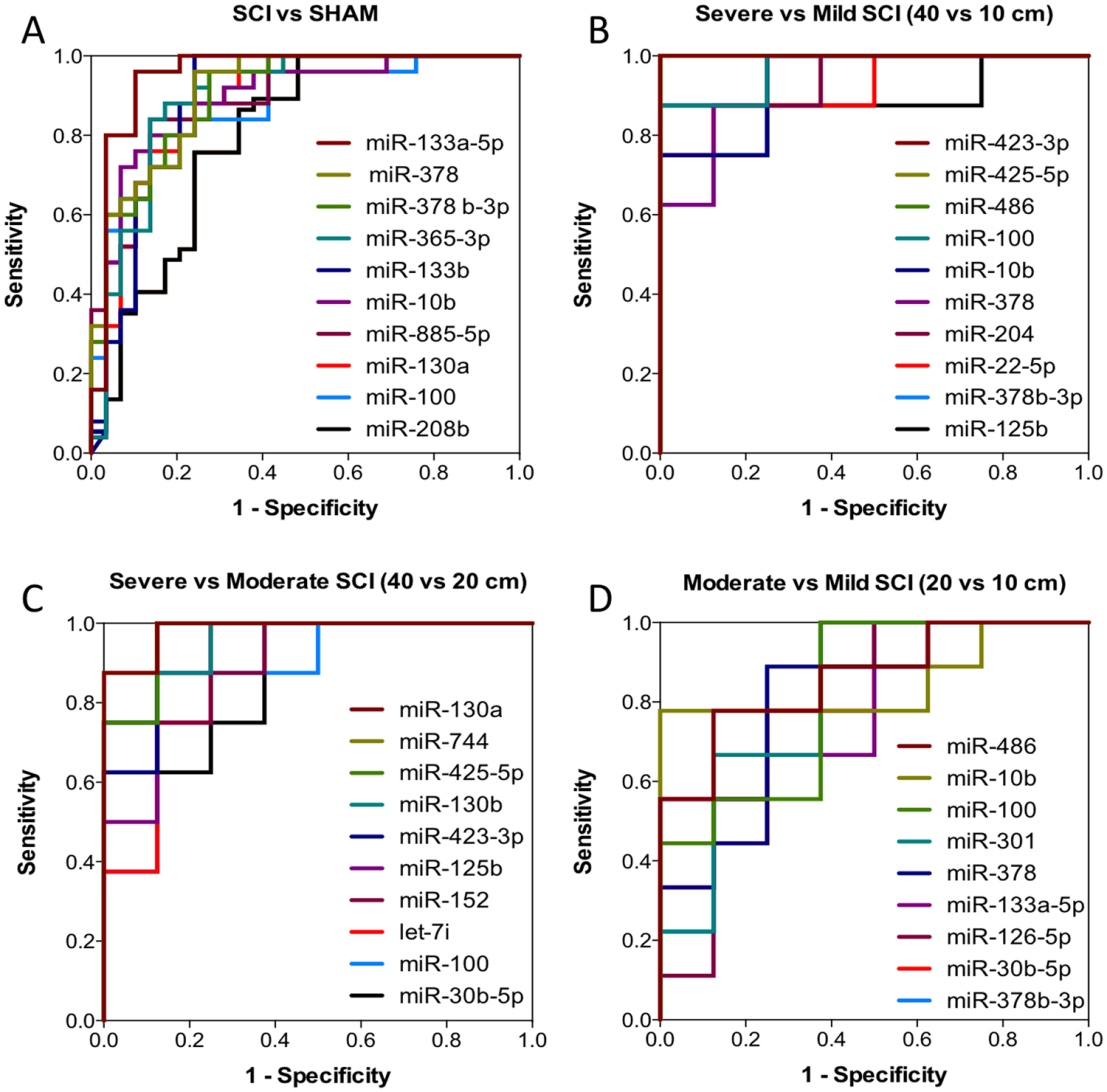
The diagnostic accuracy of significantly deregulated serum miRNAs for SCI severity. Accuracy was assessed using the ROC Curve of the 10 miRNA with the highest diagnostic accuracy (AUC) and smallest p-value, at 1 and 3 dpi, to distinguish between (**A**). SCI and SHAM (non-CNS injury control), the AUC’s were miR-133a-5p (AUC = 0.95), miR-378 (AUC = 0.91), miR-378b-3p (AUC = 0.90), miR-365-3p (AUC = 0.89), miR-133b (AUC = 0.89), miR-10b (AUC = 0.88), miR-885-5p (AUC = 0.88), miR-130a (AUC = 0.88), miR-100 (AUC = 0.88), and miR-208b (AUC = 0.87) (**B**). Severe and mild SCI (40 vs 10 cm), the AUC’s were miR-423-3p (AUC = 1.00), miR-425-5p (AUC = 1.00), miR-486 (AUC = 1.00), miR-100 (AUC = 0.97), miR-10b (AUC = 0.94), miR-378 (AUC = 0.94), miR-204 (AUC = 0.92), miR-22-5p (AUC = 0.92), miR-378b-3p (AUC = 0.92), and miR-125b (AUC = 0.91) (**C**). Severe and Moderate SCI (40 vs 20 cm), the AUC’s were miR-130a (AUC = 0.98), miR-744 (AUC = 0.98), miR-425-5p (AUC = 0.97), miR-130b (AUC = 0.95), miR-423-3p (AUC = 0.95), miR-125b (AUC = 0.92), miR-152 (AUC = 0.92), let-7i (AUC = 0.89,), miR-100 (AUC = 0.88), and miR-30b-5p (AUC = 0.88) (**D**). Moderate and Mild SCI (20 vs 10 cm), the AUC’s were miR-486 (AUC = 0.86,), miR-10b (AUC = 0.85), miR-100 (AUC = 0.82), miR-301 (AUC = 0.82), miR-378 (AUC = 0.81), miR-133a-5p (AUC = 0.79), miR-126-5p (AUC = 0.79), miR-30b-5p (AUC = 0.79), and miR-378b-3p (AUC = 0.79).

To determine the potential of specific miRNA genes to diagnose SCI and determine injury severity, receiver operator characteristic (ROC) curves were generated to calculate the area under the curve (AUC). Based on the correlation results (Supplementary Figures 4 and 5), the levels of each miRNA at 1 and 3 dpi were used to determine how well they could classify severity of injury. Using the list of dysregulated miRNA (Supplementary Table 2), the 10 miRNAs that showed the greatest diagnostic accuracy (and smallest p-value) in each pair of comparisons (by AUC) are shown in Fig. 8. Specifically, in differentiating SCI from SHAM groups, miR-133a-5p^25^ (AUC = 0.95, p < 0.0001), miR-378^26^ (AUC = 0.91, p < 0.0001), miR-378b-3p^26^ (AUC = 0.90, p < 0.0001), miR-365-3p^27^ (AUC = 0.89, p < 0.0001), miR-133b^28, 29^ (AUC = 0.89, p < 0.0001), miR-10b^30^ (AUC = 0.88, p < 0.0001), miR-885-5p (AUC = 0.88, p < 0.0001), miR-130a (AUC = 0.88, p < 0.0001), miR-100^26, 31^ (AUC = 0.88, p < 0.0001), and miR-208b^32^ (AUC = 0.87, p < 0.0001) showed the highest AUC values, some of which have been shown to have alterations after CNS injuries. In distinguishing severe from mild SCI, miR-423-3p (AUC = 1.00, p = 0.0008), miR-425-5p (AUC = 1, p = 0.0008), miR-486^33^ (AUC = 1, p = 0.0008), miR-100^26, 31^ (AUC = 0.97, p = 0.0016), miR-10b^30^ (AUC = 0.94, p = 0.0033), miR-378^26^ (AUC = 0.94, p = 0.0033), miR-204^34^ (AUC = 0.92, p = 0.0046), miR-22-5p (AUC = 0.92, p = 0.0046), miR-378b-3p^26^ (AUC = 0.92, p = 0.0046), and miR-125b^35^ (AUC = 0.91, p = 0.0063) showed the highest AUC values some of which have been shown to have alterations after CNS injuries. In distinguishing severe from moderate SCI, miR-130a^36^ (AUC = 0.98, p = 0.0011), miR-744^37^ (AUC = 0.98, p = 0.0011), miR-425-5p (AUC = 0.97, p = 0.0016), miR-130b (AUC = 0.95, p = 0.0023), miR-423-3p (AUC = 0.95, p = 0.0023), miR-125b^35^ (AUC = 0.92, p = 0.0046), miR-152^26^ (AUC = 0.92, p = 0.0046), let-7i (AUC = 0.89, p = 0.0087), miR-100^26, 31^ (AUC = 0.88, p = 0.011), and miR-30b-5p (AUC = 0.88, p = 0.011) showed the highest AUC values some of which have been shown to have alterations after CNS injuries. Finally, in distinguishing moderate from mild SCI, miR-486^33^ (AUC = 0.86, p = 0.012), miR-10b^30^ (AUC = 0.85, p = 0.016), miR-100^26, 31^ (AUC = 0.82, p = 0.026), miR-301 (AUC = 0.82, p = 0.026), miR-378^26^ (AUC = 0.81, p = 0.034), miR-133a-5p^25^ (AUC = 0.79, p = 0.043), miR-126-5p (AUC = 0.79, p = 0.043), miR-30b-5p (AUC = 0.79, p = 0.043), and miR-378b-3p^26^ (AUC = 0.79, p = 0.043) showed the highest AUC values, some of which have been shown to have alterations after CNS injuries.

## Discussion

This study represents the first description of the temporal miRNA patterns in serum using a large animal (pig) model of SCI in which these molecular assays were corroborated with behavioural and histological analysis. We have identified a set of novel miRNA in *Sus scrofa* and our results suggest that the miRNA quantities, as measured by a next generation sequencing assay, are proportional to injury severity in the serum of pigs. Analysis of serum miRNA revealed a global increase in miRNA with the greatest changes in number and magnitude of miRNA correlating with injury severity, reflecting a substantial alteration in the post-transcriptional regulatory environment. SHAM laminectomy showed similar profiles to mild injury, which is consistent with the findings of De Biase *et al*.^38^. Previous studies have highlighted the significant alteration of miRNA in central nervous system (CNS) disorders and have shown the potential of miRNA to act as markers of neurologic dysfunction^23, 26, 39–42^.

We have shown that miRNA levels at 1 and 3 dpi significantly correlate with functional and histological outcome measures. Interestingly, miRNA levels at 3 dpi correlate with PTIBS scores (r = −0.750, p = 0.0008) and spared tissue (r = −0.833, p < 0.0001) at 12 wpi nearly as well as the force by which the injury was induced (r = −0.934 p < 0.0001, r = −0.959, p < 0.0001, respectively). Our results indicate that animals with higher levels of serum miRNA at 1 and 3 dpi had worse functional outcomes 12 wpi, as measured by PTIBS, and had more cellular damage, as measured by spared tissue. These results represent an important finding: that the detection of serum miRNA at 1 or 3 dpi is nearly as predictive of 12-week post-injury functional recovery and cellular damage as the force of contusive injury itself, which is the main determinant of injury severity in this type of controlled, preclinical experimental study. The potential for having such an early objective predictor of long-term recovery after acute SCI is very relevant for clinical trials of novel therapies, as the variability in spontaneous recovery based on an early neurologic assessment (when such an assessment can actually be done) is very high. Our preclinical data suggests that serum miRNAs have promise as objective biomarkers of acute spinal cord injury that can predict long-term outcome.

The mechanisms involved in the pathogenesis of spinal cord injury (SCI) include a primary mechanical injury (impact) and a secondary injury induced by multiple subsequent biological processes, including a local inflammatory response, cytotoxicity, apoptosis, and demyelination^43–45^. In addition to local inflammation, a systemic inflammatory response, inducing organ damage, has been shown to occur following SCI^46^. Although altered gene expression significantly contributes to the pathogenesis of secondary SCI^44, 45^, the regulatory networks that control it are not well understood. An aspect of the complex nature of secondary SCI could be derived from gene regulation by miRNA^17, 39, 40^. MiRNA, as potential indicators of a pathological state, are carried to the periphery and are appealing candidates for monitoring central nervous system pathophysiology related to SCI.

In order to establish miRNA biomarkers that are representative of acute injury within the spinal cord, the “tissue” that is in closest proximity to the injured cord, and which is obtainable in human patients, is the CSF. Damage to the spinal cord releases proteins, metabolites, and nucleic acids into the CSF^12, 16^ and we have previously identified several inflammatory and structural proteins related to injury severity within the CSF of human patients with SCI^47^. While CSF might be the most logical sampling site, logistically the serum samples represent a less invasive biological fluid that is significantly easier to obtain. In this study, we intended on providing strong evidence in support of CSF borne miRNAs that were detectable in serum and showed a severity-dependent pattern of expression. Unfortunately, the profiling of CSF miRNAs proved to be unsuccessful in a majority of pig CSF samples. Therefore, after verifying that the miRNAs seen in CSF are also observed in serum with significant correlation between their expression levels, we focused on identifying markers of injury severity in the serum.

MiRNA have been previously investigated in the setting of SCI in a number of rodent studies. Hachisuka *et al*.^48^ showed that several miRNAs increased following SCI in a severity-dependent fashion in mouse serum. In a microarray study using a contusion model of SCI in rats, the levels of over 35% of the miRNA expressed in spinal cord tissue were significantly altered within 7 dpi^26^. A separate study of changes in gene expression within different spinal cord regions showed greater changes in moderate and severe injuries, compared to mild and SHAM injury. As we observed in pig, the expression of some rodent genes was proportional to injury severity^38^.

The pig model is a large animal model of SCI and has several advantages over rodent models of SCI. In addition to the anatomical similarities between pig and human, measuring temporal changes in neurochemical markers is possible in pig because of the ability to serially collect samples over time due to the larger CSF-filled subarachnoid space in pigs compared to rodents. In addition, the miRNA profile of pigs is more conserved with humans compared to rodents^19^, with 45% of the known pig miRNA having human orthologs, compared to only 16% of the known rat miRNA^49^. Together, these factors make the pig a more relevant model, with regards to clinical translation, for studying the changes in miRNA following SCI in patients.

The mechanisms that underlie the global, severity-dependent increase in miRNA abundance we observed are not yet clear. To reiterate, all animals in our study (SHAM included) underwent the same surgical approach, the same extent of laminectomy, and the same sample acquisition protocols – the only difference between groups was the height from which the weight was dropped onto the exposed spinal cord. Therefore, the differences in serum miRNA expression between groups cannot be explained by a difference in *surgical* trauma, since the surgeries themselves were identical, but only by a more severely injured spinal cord. Similarly, the increase seen in the SCI animals versus SHAM control animals cannot be explained by surgical insult because the SHAM animals underwent the identical surgical exposure and laminectomy and only differed by the absence of the contusion injury. The most parsimonious explanation is that the actual traumatic SCI is directly or indirectly driving up the global concentration of miRNA within serum. In previous studies, a number of structural and inflammatory markers were reported as biomarkers for SCI severity^9, 12, 16, 47^. These likely arose from acute damage to the spinal cord itself. Recently, circulating cell-free DNA (cfDNA) has been investigated as a potential biomarker for TBI, where cfDNA released from injured cells in the brain contain specific methylation patterns that can be detected in the blood through targeted DNA sequencing^50^.

In light of these caveats, our observations are consistent with the observed increase in miRNA reflecting active, biogenic upregulation of miRNA systemically. While the miRNA accumulation we observe could be specific, it may also reflect downstream effects of a systemic inflammatory response that is proportional to the severity of injury. A systemic inflammatory response in relation to SCI has been documented^46^. Additionally, Bao *et al*.^51^ showed evidence for the activation of circulating inflammatory cells after spinal cord injury that potentially damage tissues outside the spinal cord. With an increased systemic inflammatory response, and increased cell turnover, there may be elevated levels of cellular miRNA in circulation.

Interestingly, Yunta, Nieto-Diaz^35^ have shown a global *downregulation* of miRNA within spinal cord tissue of rodents following SCI. The authors suggest the changes could be due to the death of specific cell types within the spinal cord. We would hypothesize that this downregulation of miRNA within spinal cord tissue could contribute to the global upregulation we observe in fluid – the acute damage to the spinal cord causes a massive cell death event, releasing cellular contents into circulation, ie: less miRNA in tissue, more in circulation. Studies in TBI^50^, liver injury^52^, and SCI^9, 16^ have shown that acute damage to the brain, liver, or spinal cord, resulting in cell death, produces measurable biomarkers in the blood or CSF that can be traced back to the tissue of origin. The fact that the spinal cord injury itself, and not the SHAM surgery, inflicts a rise in serum miRNA in a severity dependent pattern provides a specific, promising biomarker for injury severity.

There are a number of limitations worth discussing with regards to this study. Firstly, global upregulation of miRNA seems to be a strong indicator of injury severity and correlates well with 12 wpi outcome, however the overall cumulative global expression was not found to be statistically different between the mild and sham groups (Fig. 7A,B). This suggests that mild spinal cord injury may be difficult to detect based on overall miRNA expression. While this may be the case, there were 4 miRNA found to be differentially expressed in the mild SCI group (3 of which were also dysregulated in other SCI groups), but not in the SHAM group (Fig. 5). These miRNA may provide the subtle discrimination when the differences between groups are not easily detectable and may indicate the difference between CNS injury and surgical treatment. Secondly, the numbers of replicates in this study are relatively low. Initially, six biological replicates were used in each group (24 animals). Unfortunately, sample collection was a challenging aspect of this study. We therefore selected animals in which a full set of corresponding CSF and serum at all four time points was present, resulting in four animals per group. One advantage to this porcine model of SCI, however, is the ability to serially collect samples from the same animals – to be able to investigate expression changes, over time, in the same animal. While this study describes four experimental groups, they are not mutually exclusive; each injury severity group is a gradation of the same treatment, and the results show a corresponding gradation of miRNA expression. We felt that the inclusion of four experimental severities and six replicates per group were stronger than, say, two experimental groups (SCI and control, for example) and 12 replicates in each group. This gives us confidence in the statistical power of the study, as well as in the observed effects. Thirdly, the associated costs with surgery, post-surgical care, and 12 weeks of animal housing, in addition to sample preparation costs, were also limiting. With four replicates and four time points across four treatment groups, next-generation sequencing was performed on 128 CSF and serum samples. Finally, while this study lacks the validation of specific miRNAs using PCR-based detected methods, this was a strategic decision to focus on surveying all detectable miRNAs in both CSF and serum. Our ultimate goal is in the development of a clinical and pre-clinical biomarker that can be used in both humans and our pig model. Validation of miRNAs that might be pig-specific would be premature. Following this study, we are profiling miRNA changes in the CSF and serum collected from human patients with traumatic SCI. While there is a relatively high cost associated with miRNA sequencing, the economic benefit will far outweigh the cost of sample preparation, and the ability to accurately diagnose patients will be invaluable. Once we have identified top miRNA biomarker candidates that exist in *both* human and pig biofluids, we will aim to validate these miRNA in both species and generate a panel of genes that are highly diagnostic of SCI severity, and predictive of outcome.

## Conclusions

Our study provides a comprehensive description of the changes that occur across miRNA during the early post-injury phase of acute SCI. The miRNA detected in porcine serum increased globally in an injury severity dependent manner and provides promising targets for markers of injury severity in SCI. The results from this study will guide the investigation of temporal changes in miRNA within human samples of CSF and serum, collected from patients with traumatic SCI. With the continuous emergence of new SCI therapies that are seeking validation in clinical trials, the field of SCI is in dire need of new approaches for classifying injury severity and improved methods of predicting outcome. MiRNA biomarkers are promising solutions to this bottleneck in the pipeline of developing clinically relevant therapies for spinal cord injury.

## Materials and Methods

All animal procedures were performed in accordance with the guidelines of the Canadian Council for Animal Care and approved by the University of British Columbia’s Animal Care Committee.

### **A**nimals and Experimental Design

Female Yucatan miniature pigs (*Sus scrofa)* (Sinclair Bio-resources, Columbia, MO) weighing 20–30 kg were group-housed at a large animal facility for 5 weeks prior to surgery. Animals were block-randomized into different injury severity groups, or a SHAM group. Animals in the SHAM-operated surgery group (n = 4) received an identical laminectomy surgery as in the injured groups, but no weight drop injury or compression to the spinal cord. 3 different injury severities were induced by dropping a 50 g weight onto the exposed spinal cord from a height of 40, 20, or 10 cm (n = 4 per group), followed by 5 minutes of compression with a 150 g weight. Samples of cerebrospinal fluid (CSF) and serum were collected 15 minutes before injury (baseline, BSL) and then again at 1, 3, and 5 dpi. Total RNA was isolated from samples of CSF and serum, and using next-generation sequencing, we profiled known micro RNA (miRNA), identified novel miRNA, and compared the time-course miRNA profiles between CSF and serum over 5 days, following injury.

### Porcine Model of Traumatic SCI

Surgical procedures for spinal cord injury (SCI) and post-operative care were performed as previously described^20, 53^. Using anatomic landmarks, the T9, T10, and T11 pedicles were cannulated and instrumented with screws (Select^TM^ Multi Axial Screw, Medtronic, Minneapolis, MN). After the T10 laminectomy was performed, the weight drop device was rigidly secured to the pedicle screws and positioned so that the impactor (weight: 50 g) would fall directly on the exposed dura and spinal cord at T10. The tip of the impactor (diameter: 9.53 mm) was instrumented with a load cell (LLB215, Futek Advanced Sensor Technology, Irvine, CA, USA) to record the force at impact. Immediately following the contusion injury, compression was applied by placing a 100 g mass on top of the impactor (150 g total) for 5 minutes.

### CSF Collection

The technique for serially collecting CSF samples in our pig model post-injury has been previously described^53^. In summary, CSF collection was achieved using a 19 gauge epidural catheter (Perifix epidural catheter set; Braun Medical Inc., PA) inserted into the intrathecal space with the catheter tip resting approximately 8 cm caudal to the injury site. A total of 1 mL of CSF was collected at baseline (BSL), 15 minutes before injury, and at 1, 3, and 5 dpi. Immediately after collection, CSF samples were centrifuged at 1,000 g for 10 minutes at room temperature. The CSF supernatant was aliquoted into a 2.0 mL RNAse free centrifuge tube (Eppendorf^TM^, ON), immediately frozen on dry ice, and stored at −80 °C for RNA isolation.

### Blood/Serum Collection

Blood collection was performed by inserting an 8F Groshong catheter (Bard Access Systems, Inc, Salt Lake City, UT) in the left external jugular vein. This was connected to a low volume titanium subcutaneous access port (X-port, Bard Access Systems. Salt Lake City, UT) housed in the posterior neck region. A sterile 22-gauge Huber needle (Instech Solomon. Plymouth Meeting, PA) was used to access the port for withdrawal of blood. A total of 5 mL of whole blood was collected at BSL, and at 1, 3, and 5 dpi. To separate the serum portion, blood was allowed to incubate for 25 minutes at room temperature, and then centrifuged at 10,000 g for 5 minutes. The serum supernatant was aliquoted into a 2.0 mL RNAse free centrifuge tube (Eppendorf^TM^, ON), immediately frozen on dry ice, and stored at −80 °C for RNA isolation.

### Porcine Thoracic Injury Behaviour Analysis

To assess hindlimb functional recovery, the Porcine Thoracic Injury Behavior Scale (PTIBS) was used, as previously described^20, 53^. Briefly, four weeks prior to injury, animals were trained to walk straight along a rubber mat (4.0 m × 1.2 m) at a constant speed without stopping. Clicker training, along with a food reward, was used for motivation. Baseline behavior was obtained for each animal, one week prior to surgery: five runs were recorded with three high-definition camcorders placed 30 cm above the ground and behind the animals. Functional assessment resumed one week post-injury and continued once weekly for 12 weeks. The functional assessment footage was analyzed by two independent observers that were blinded to the biomechanical severity of spinal cord injury that was induced at the time of surgery. These observers were members of the research team, and familiar with the PTIBS scoring system, but were not directly involved in the surgical or post-operative care aspects of the project and thus were not aware of the severity of injury for each animal. The PTIBS scale ranges from no active hindlimb movements (score 1), to normal ambulation (score 10). PTIBS scores of 1–3 are characterized by “hindlimb dragging,” scores of 4–6 reflect varying degrees of “stepping” ability, and scores of 7–10 reflect varying degrees of “walking” ability.

### Histological Outcomes

At the end of the experiment (12 weeks post-injury), animals were euthanized, the spinal cord harvested, post-fixed and cryoprotected as described previously^53^. Subsequently, spinal cords were cut into 1 cm blocks centered on the site of injury, frozen on dry ice, and stored at −80 °C. Cross-sections (20 μm thick) were then cut using a cryostat. Sections were serially mounted onto adjacent silane-coated SuperFrost Plus slides (Fisher Scientific, Pittsburgh, PA) such that sections on the same slide were obtained from tissue 400 μm apart and stored at −80 °C. For differentiating grey and white matter, Eriochrome Cyanine R staining (EC) was performed with Neutral Red as a counterstain. EC-stained sections were examined and micrographs (5x objective) were taken of sections at 800 μm intervals throughout the lesion site (Zeiss AxioImager M2 microscope, Carl Zeiss Canada Ltd., Toronto, ON, Canada). The spinal cord outer perimeter, white matter, and gray matter were outlined, and the area of each was calculated using Zen Imaging Software (Carl Zeiss Canada Ltd., Toronto, ON, Canada). The spared white matter was defined as areas that exhibited dense blue staining. Spared gray matter was defined based on the color of the stains and morphology. We define intact gray matter as tissue containing normal gray matter cytoarchitecture with visible neutral red staining present. The percentages of white matter and grey matter were calculated by dividing the spared white or grey matter by the total area of the spinal cord on a given section and the sum of the two, representing “total spared tissue”.

### RNA Isolation and Sequencing

Total RNA was isolated from 1 mL of CSF or serum using the miRVana PARIS kit (ThermoFisher, Cat#: AM1556) according to the manufacturer’s instructions, incorporating the modifications of Burgos *et al*.^21^ for maximum yield. Total RNA was re-suspended in 6 µL of water and used for library preparation with Illumina’s TruSeq Small RNA Library Preparation Kit (Illumina, San Diego, CA). The following modifications were introduced to the library preparation to account for the very low amounts of input RNA in all samples: 1. Each reaction used only half of the recommended reagent amounts, and 2. The PCR cycles during library amplification were increased to 15. Libraries were individually barcoded with Illumina-provided index barcodes so that samples could later be demultiplexed. Libraries were pooled and sequenced on the Illumina MiSeq or HiSeq 2500, generating single-end 36 bp reads.

### Post-Sequencing Analysis Pipeline

Sequencing reads were processed using the Mayo Clinic’s Comprehensive analysis pipeline for miRNA sequencing data (CAP-miRSeq)^54^. The pipeline is summarized in Fig. 4A. Read quality was assessed using FastQC^55^ before and after trimming adapter sequences and low quality 3′ bases. Adapter sequences were trimmed with Cutadapt^56^ and reads shorter than 17 nt discarded. Trimmed reads were first aligned using Bowtie^57^ where the pipeline conducts two alignment processes: one used internally for miRDeep2 to quantify and predict novel miRNA and ano3ther for all RNA quantification and data visualization. The CAP-miRSeq pipeline generates a summary for each sample’s alignment statistics and number of miRNA detected with reports of raw counts for known miRNA of all samples in matrix format. Samples with a total sum of mapped miRNA read counts less than 50,000 reads per sample were removed from analysis. This 50,000 read threshold was determined based on the Spearman correlations of randomly selected subsets of reads to the total number of reads in a sample (Supplementary Figure 1).

### Statistical analysis

Adjustments for differences in sequencing depth were made using the number of aligned reads, and the counts were adjusted to “miRNA reads, per million aligned reads”. In other words, the number of miRNA reads proportional to all other small RNAs.

$$Normalized\,MicroRNA\,{Reads}=\frac{Raw\,MicroRNA\,{Reads}}{Aligned\,{Reads}}\times \mathrm{1,000,000}$$

This normalization accounts for technical differences in library sizes, where a larger library will be associated with higher numbers of miRNA counts, *as well as* aligned read counts. To ensure this proportion is unchanged due to technical differences, 3 biologically different samples were sequenced with differing concentrations, on different devices, and different dates. While the raw number of reads and number of aligned reads varied greatly due to different concentrations being loaded, the ratio of raw miRNA reads to aligned reads remained identical. This provided confidence in the ability of this normalization method to account for technical differences in pipetting, library size, or sequencing efficiency.

Since most normalization strategies rely on the assumption that most genes are not differentially expressed^58^, we used an alternative method of normalization. The rationale for this normalization was designed to accommodate differences in global levels of miRNA levels^59–62^. This method is well suited for our NGS dataset, which includes reads for the entire set of miRNA, rRNA, yRNA, tRNA, snoRNA, and other R-Fam members that are captured during library preparation. Statistically significant miRNA were determined using multiple t-tests, while correcting for multiple comparisons using the Benjamini-Hochberg method with adjusted p-values lower than 0.05. Differences in total miRNA counts were determined using two-way analysis of variance (ANOVA) after assessing for distribution and variance. MicroRNA with statistically significant AUC of the ROC curve were determined after correcting for multiple testing using the Benjamini-Hochberg method with adjusted p-values lower than 0.05.

### Data access

The data has been submitted to the Sequence Read Archive (SRA) database in BioProject accession number [SRA: SRP082514].

### Declarations

#### Ethics approval

All animal procedures were performed in accordance with the guidelines of the Canadian Council for Animal Care and approved by the University of British Columbia’s Animal Care Committee.

## Electronic supplementary material

Supplementary Figures & Tables
