# Supplementary material for: Serum MicroRNAs Reflect Injury Severity in a Large Animal Model of Thoracic Spinal Cord Injury
Source: Sci Rep. 2017 May 3;7:1376. doi: 10.1038/s41598-017-01299-x (PMC5431108; doi:10.1038/s41598-017-01299-x)

Supplementary Figures and Tables

Serum MicroRNA Reflects Injury Severity in a Large Animal Model of Thoracic Spinal Cord Injury

Seth Tigchelaar[1], Femke Streijger[1], Sunita Sinha[2], Stephane Flibotte[2], Neda Manouchehri[1], Kitty So[1], Katelyn Shortt[1], Elena Okon[1], Michael Rizzuto[1], Ivana Malenica[3], Amanda Courtright[3], Andrew Eisen[4] Kendall Van Keuren-Jensen[3], Corey Nislow[2], Brian K. Kwon[1, 5, *]

[1]International Collaboration on Repair Discoveries (ICORD), University of British Columbia, Vancouver, British Columbia, Canada;

[2]Faculty of Pharmaceutical Sciences, University of British Columbia, Vancouver, British Columbia, Canada;

[3]Translational Genomics, Phoenix, Arizona, United States of America;

[4]Acorda Therapeutics, Ardsley, New York, United States of America;

[5] Vancouver Spine Surgery Institute, Department of Orthopedics, University of British Columbia, Vancouver, British Columbia, Canada

[*]**Corresponding Author (and for Reprints):**

Brian K. Kwon, MD, PhD, FRCSC

Canada Research Chair in Spinal Cord Injury

Professor, Department of Orthopaedics, University of British Columbia

6th Floor, Blusson Spinal Cord Centre, Vancouver General Hospital

818 West 10th Avenue, Vancouver, BC, CANADA, V5Z 1M9

PH: 604-875-5857

FX: 604-875-8223

E-mail: brian.kwon@ubc.ca

Seth Tigchelaar	s.tigchelaar@alumni.ubc.ca	Ph: 604-675-8837, Fax: 604-675-8849

Femke Streijger	streijger@icord.org	Ph: 604-675-8837, Fax: 604-675-8849
Sunita Sinha	Sunita.sinha@ubc.ca	Ph: 604-827-0310, Fax: 604-675-8849
Stephane Flibotte	sflibotte@gmail.com	Ph: 604-827-0310, Fax: 604-675-8849
Neda Manouchehri	nedamanouchehri@gmail.com	Ph: 604-675-8837, Fax: 604-675-8849
Kitty So	Kitty.ubc@gmail.com	Ph: 604-675-8837, Fax: 604-675-8849
Katelyn Shortt	Katelyn.shortt@gmail.com	Ph: 604-675-8837, Fax: 604-675-8849
Elena Okon	okon@icord.org	Ph: 604-675-8837, Fax: 604-675-8849
Michael Rizzuto	Michael.rizzuto@alumni.ubc.ca	Ph: 604-675-8837, Fax: 604-675-8849
Ivana Malenica	imalenica@tgen.org	Ph: 602-343-8400, Fax: 602-343-8411
Amanda Courtright	acourtright@tgen.org	Ph: 602-343-8400, Fax: 602-343-8411
Andrew Eisen	aeisen@acorda.com	Ph: 914-326-5058, Fax: 914-606-9634
Kendall Van Keuren-Jensen	kjensen@tgen.org	Ph: 602-343-8400, Fax: 602-343-8411
Corey Nislow	corey.nislow@ubc.ca	Ph: 604-827-0310, Fax: 604-675-8849
*Brian K. Kwon	brian.kwon@ubc.ca	Ph: 604-875-5857, Fax: 604-875-8223

Supplementary Table 1. Novel miRNA. To be listed, the potential miRNA must be present in at least 30% of either Serum or CSF samples, and have more than 5 counts on average across all samples. Column one through three contains the location of the sequence predicted by miRDeep2 for the potential mature miRNA. Column four is the percentage of serum samples in which the miRNA was detected and column five is the percentage of CSF samples in which the miRNA was detected. Column six represents the total percentage of samples in which the miRNA was detected.

miRNA Location						
Chr	Start	Stop	Mature Sequence	% of serum Samples	% of CSF Samples	% of Total Samples
chr15	133957559	133957616	gcgacgagccccucgcacaaaccggaccugagcguuuuguucguucggcucgcguga	76.53	50.00	71.31
chr6	870006	870063	cggugcgggucggggcggggcggacuguccucagugcgccccgggcgggucgcgccg	75.51	50.00	70.49
chrX	39824716	39824776	gccaguuacuuccgcucugauggaucuauuuccauuucuagcggcggcgacucuggacuc	71.43	45.83	66.39
chr12	37079098	37079158	gcucugacuuuauugcacuacuguacuuuacagcuagcagugcaauaguauugucaaagc	71.43	41.67	65.57
chr5	66193719	66193780	ucuuccagcacaguguuggauggucuaauagugaagcuccuaacacugucugguaaagaug	67.35	50.00	63.93
chr13	24885255	24885316	uucaaguaacccaggauaggcugugcaggucccaaggggccuauucuugguuacuugcacg	68.37	41.67	63.11
chr10	6017465	6017551	guccgaugguaguggguuaucagaacuuauuaacguuagugucucuaaaguugguauacaacccccc ccacugcuaaauuugacug	64.29	50.00	61.48
chrX	48640827	48640885	uacccauugcauaucggaguugugaauucucaaagcaccuccuaugugcaugguuuac	57.14	33.33	52.46
chr16	51742262	51742327	gcagagucaagccugguuagcacuuggaugggagaccgucugggcauaccgggugcuguaggcuu	55.10	25.00	49.18
chr7	55687381	55687443	ucugguucugugacccugccuauuugcccuucagccagguaagagcaucugaaccaggggu	46.94	16.67	40.98
chr5	30185306	30185368	gugccugggucccccuccccagagguucaaauuuaauugcucugagaugugaccugggcaucc	38.78	37.50	38.52
chr3	42401527	42401607	cugauguggcggugggcaacgugagggcagccagcaccugugagguugguggugcugacguuggcucu gcgaggucggcu	39.80	25.00	36.89
chr7	69920245	69920312	cggcggccgggggcgccggaggguccacugagcuuccuccccacccuccccgccuccucucgccgcc	37.76	25.00	35.25
chr10	50066936	50066995	ggcgggcccacgggggcccccggugagcccgggccgccgggccccgugggcccccgg	30.61	37.50	31.97
chr12	35722001	35722067	cccuugaugaucguucuccucucucugguugagagaggcagagggagagaacaccgucugaguggu	27.55	33.33	28.69

Supplementary Figure 1. Spearman Correlation (green) of subsets of randomly selected reads to a total of 1.7 million reads, starting with 1,000 reads and incrementally increasing by thousands to 300,000. Number of miRNA detected (blue) using randomly selected reads, starting with 1,000 reads and incrementally increasing by thousands to 300,000.

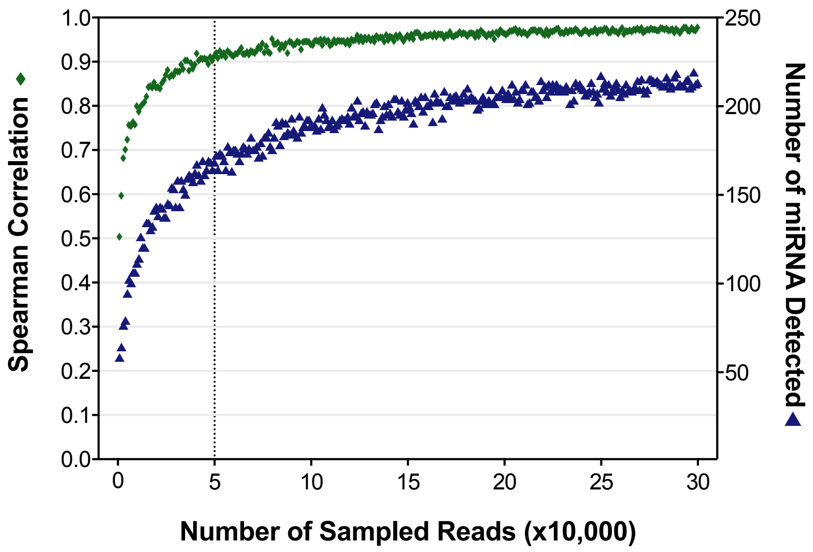

Supplemental Figure 2. Correlation of serum and CSF miRNA counts for all detected genes.

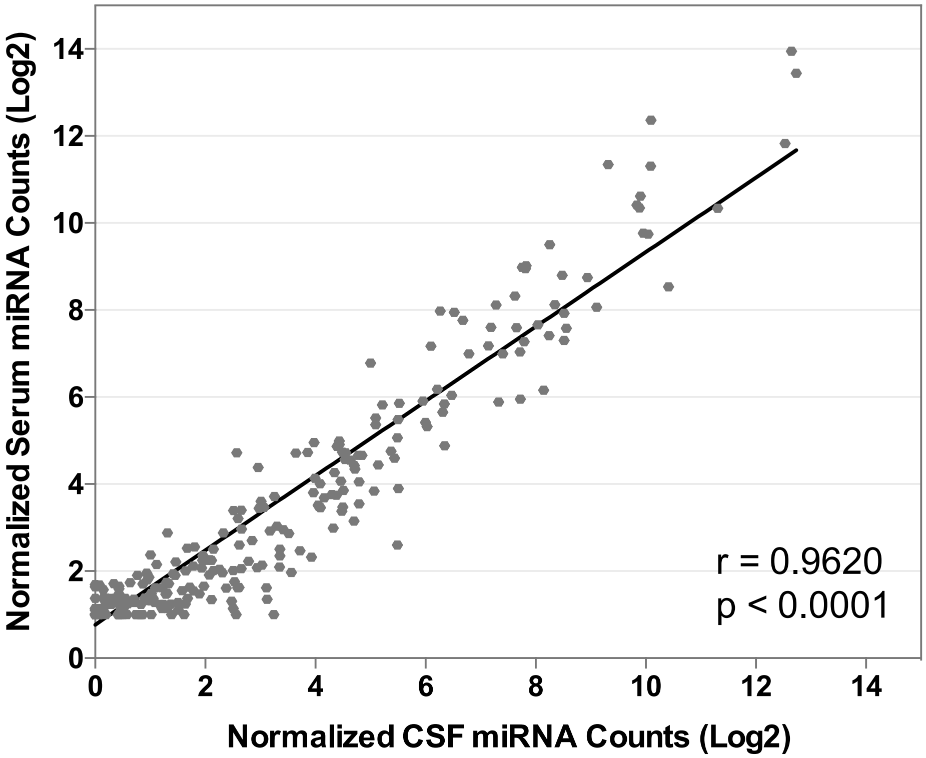

Supplementary Figure 3. Effect of Injury Severity on Global miRNA Expression. Raw data showing the global miRNA expression at BSL, 1, 3, and 5 dpi in each of the A. 40 cm, B. 20 cm, C. 10 cm, and D. SHAM groups. Datapoints represent the average read counts for the top 100 miRNA genes for n=4 animals per group and time point (see Table 1).

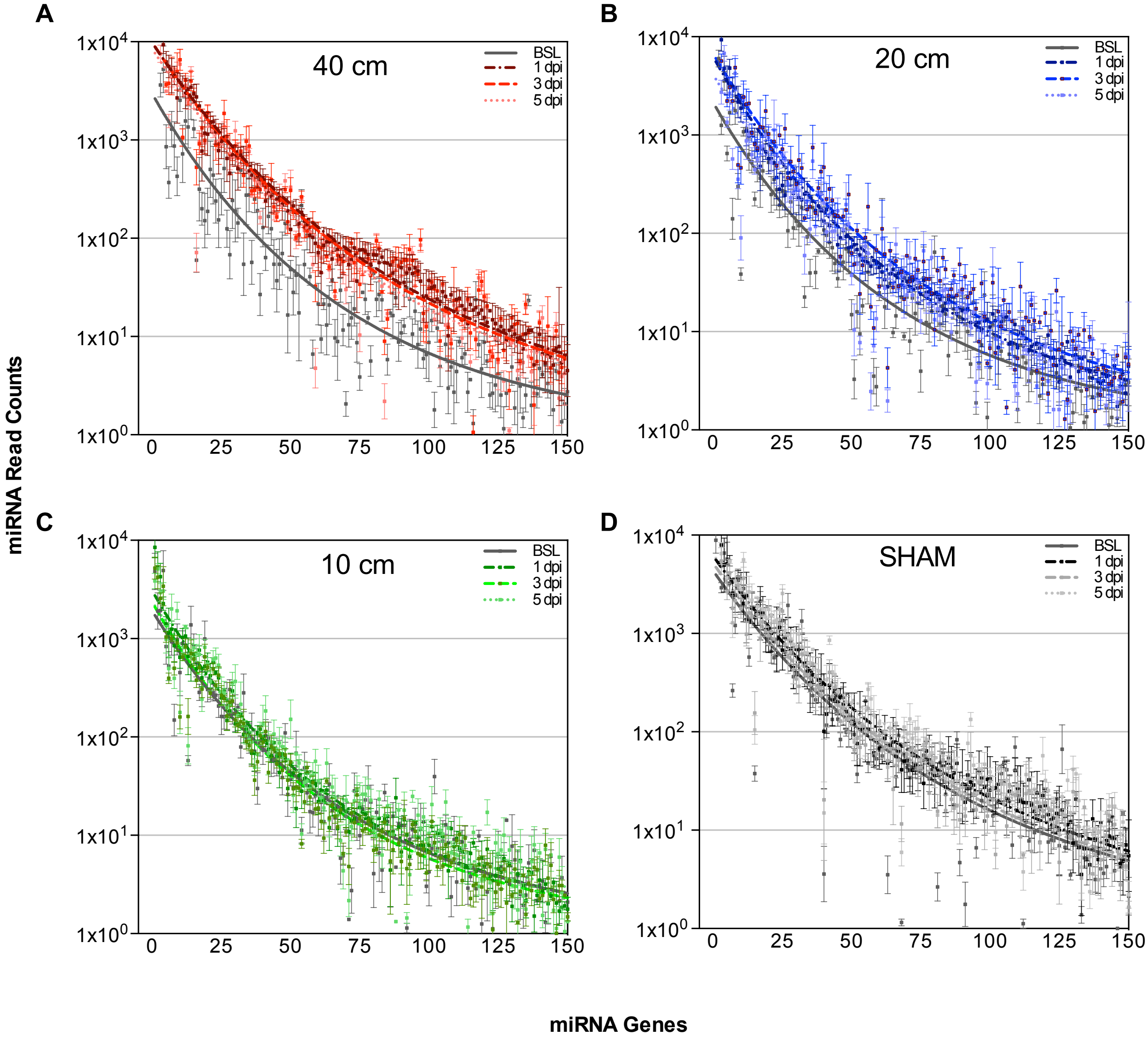

Supplementary Table 2. Table of deregulated serum miRNA. Log_2 Fold-Change values, relative to baseline after severe (40 cm, red), moderate (20 cm, blue), mild (10 cm, green) injury, or SHAM surgery (Non-CNS Injury control, grey). Associated p-values are denoted as * ($p < 0.05$), ** ($p < 0.005$), *** ($P < 0.0005$). DPI, days post injury; L2FC, Log_2 Fold-Change. Only time points with significantly deregulated miRNA are shown. Total numbers of deregulated miRNAs are shown at bottom.

	Severe SCI (40 cm)						Moderate SCI (20 cm)				Mild SCI (10 cm)		SHAM (SCI Control)			
	1 dpi		3 dpi		5 dpi		1 dpi		3 dpi		1 dpi		1 dpi		5 dpi	
Mature miRNA	L_2FC	p	L_2FC	p	L_2FC	p	L_2FC	p	L_2FC	p	L_2FC	p	L_2FC	p	L_2FC	p
let-7a			1.45	*	1.44	*										
let-7d-3p					1.74	*										
let-7d-5p					1.73	*										
let-7e			2.37	**	2.31	**										
let-7f			1.40	*	1.39	*										
let-7g			1.39	*	1.35	*										
let-7i					1.27	*										
miR-1	7.03, **	**														
miR-100							1.38	**	1.23	**						
miR-10b											1.09	**				
miR-125b	1.29	**														
miR-126-3p			1.96	*	1.90	*										
miR-126-5p									1.26	*						
miR-127	2.70	***	2.22	**			2.19	***			1.69	***	2.76	***		
miR-128	1.79	**	1.72	**	1.53	*	1.49	**			1.31	**				
miR-1285							0.72	**	0.55	**	0.68	**				
miR-130a	1.58	*	1.54	*	1.55	*	1.33	*	1.40	**						
miR-130b			1.41	*	1.42	*										
miR-133a-3p	1.93	***	1.53	**			1.89	***	1.48	**	1.70	***	2.00	***		
miR-133a-5p	3.82	***	3.78	***	3.00	**	2.56	***	2.27	***						
miR-133b	15.20	***	11.88	***			12.29	***	8.68	***	12.05	***	9.33	***		
miR-142-3p			1.87	**	1.77	*										
miR-142-5p			1.44	*	1.43	*										
miR-143-3p							1.26	**								

**miR-144**					2.93	*										
**miR-145-3p**	1.82	*														
**miR-145-5p**	1.89	**														
**miR-1468**									1.31	**					1.39	***
**miR-148b-3p**			3.24	*	3.20	*										
**miR-149**	1.41	**	1.41	**	1.34	**										
**miR-150**			1.47	**	1.49	*										
**miR-152**			1.63	**	1.51	*										
**miR-15a**	2.10	*	1.96	*	2.01	**										
**miR-204**	6.38	**														
**miR-208b**	5.85	***	3.33	***			5.50	***			4.85	**	7.43	**		
**miR-216**	3.67	***														
**miR-22-3p**	1.18	*	1.16	*			1.21	**								
**miR-22-5p**	3.26	**	3.20	**	2.57	**										
**miR-221-3p**			1.43	***	1.42	**										
**miR-222**			1.67	**	1.73	**										
**miR-23b**			3.27	*	3.10	**										
**miR-26a**			1.55	*	1.53	*										
**miR-27a**	1.50	*	1.41	*												
**miR-27b-3p**	1.29	*					1.20	**	1.11	**						
**miR-29c**	4.24	*														
**miR-301**			2.33	**	2.30	**										
**miR-30a-3p**									1.33	**						
**miR-30a-5p**							1.18	*	1.17	**						
**miR-30b-5p**			1.60	*												
**miR-365-3p**	2.78	*					1.59	*	1.57	*	1.48	***				
**miR-378**	1.46	**	1.39	**	1.24	*	1.51	***	1.40	**	1.32	**	1.37	***		
**miR-378b-3p**	2.26	**	2.43	***	1.96	**	2.43	**	2.38	**					2.26	**
**miR-423-3p**			1.12	*												
**miR-425-3p**			2.08	*												
**miR-425-5p**			1.31	*	1.28	**										
**miR-4331**	0.79	**	0.78	*	0.63	**										
**miR-450b-5p**	2.30	***														

**miR-451**		1.36 *	1.32 *					
**miR-486**		1.17 *			1.19 **			
**miR-574**	1.51 **	1.48 ***	1.46 **					
**miR-7134-3p**		1.56 *	1.57 **					
**miR-7139-5p**			1.65 **		1.64 *			
**miR-744**			1.35 *					
**miR-885-5p**	8.09 **	6.50 *						
**miR-98**		1.86 *	1.81 *					
**miR-9841-3p**		1.78 **	1.73 **					
**Total**	**27**	**42**	**37**	**16**	**16**	**9**	**5**	**2**

Supplementary Figure 4. Correlations between total miRNA expression and Porcine Thoracic Injury Behaviour Scores. Correlation between 12 wpi PTIBS scores and the total miRNA counts at. **A.** Baseline before injury (BSL), **B.** 1 dpi, **C.** 3 dpi, and **D.** 5 dpi. The global upregulation in miRNA at 1 and 3 dpi is correlated strongly with the behavioral recovery at 12 weeks post-injury.

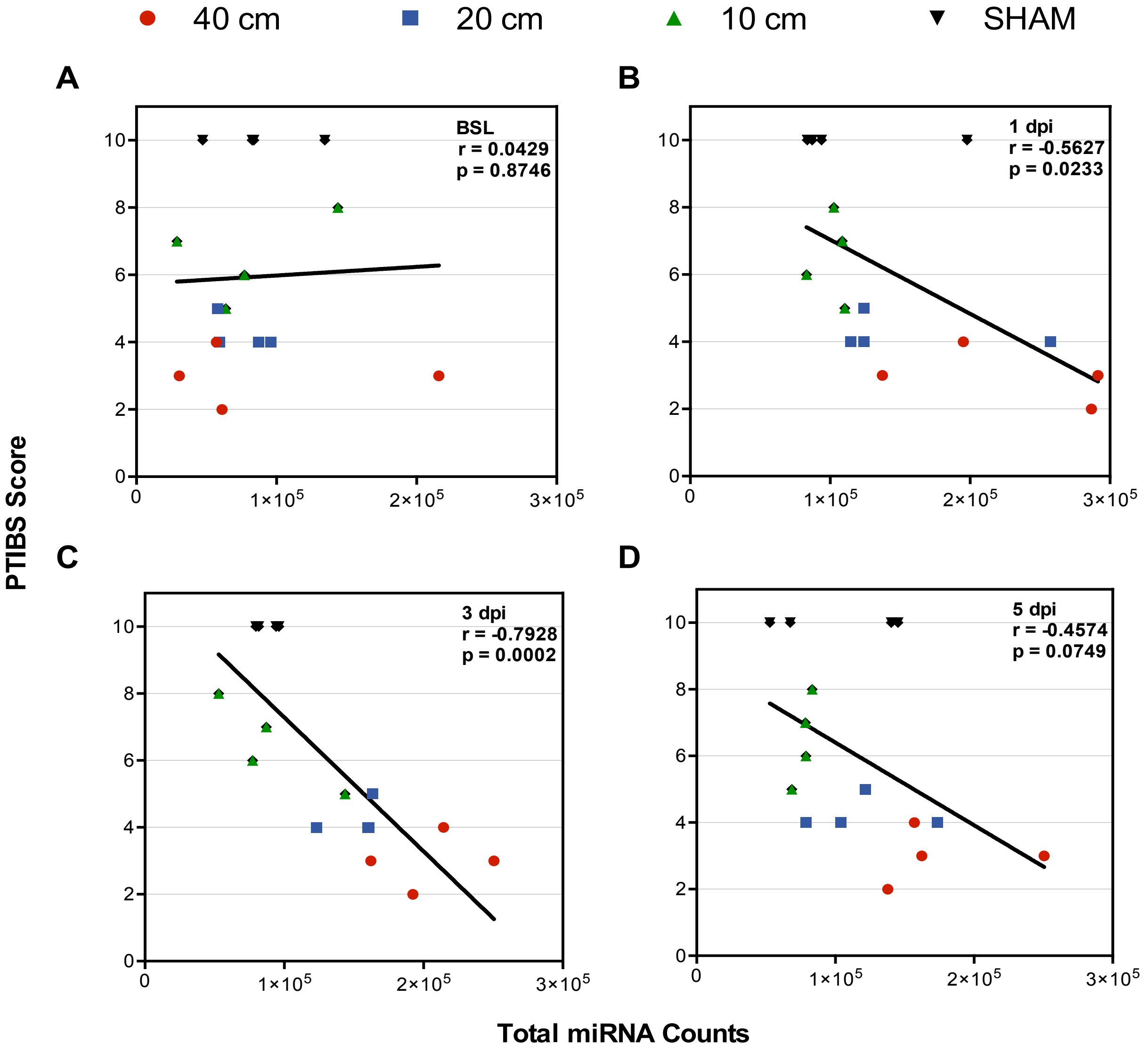

Supplementary Figure 5. Correlations between total miRNA expression levels and total percent spared tissue. Correlation between total percent spared tissue and the total miRNA counts at **A.** baseline before injury (BSL), **B.** 1 day post-injury (dpi), **C.** 3 dpi, and **D.** 5 dpi. The global upregulation in miRNA is correlated strongly with the extent of tissue damage at 1 and 3 dpi in particular.

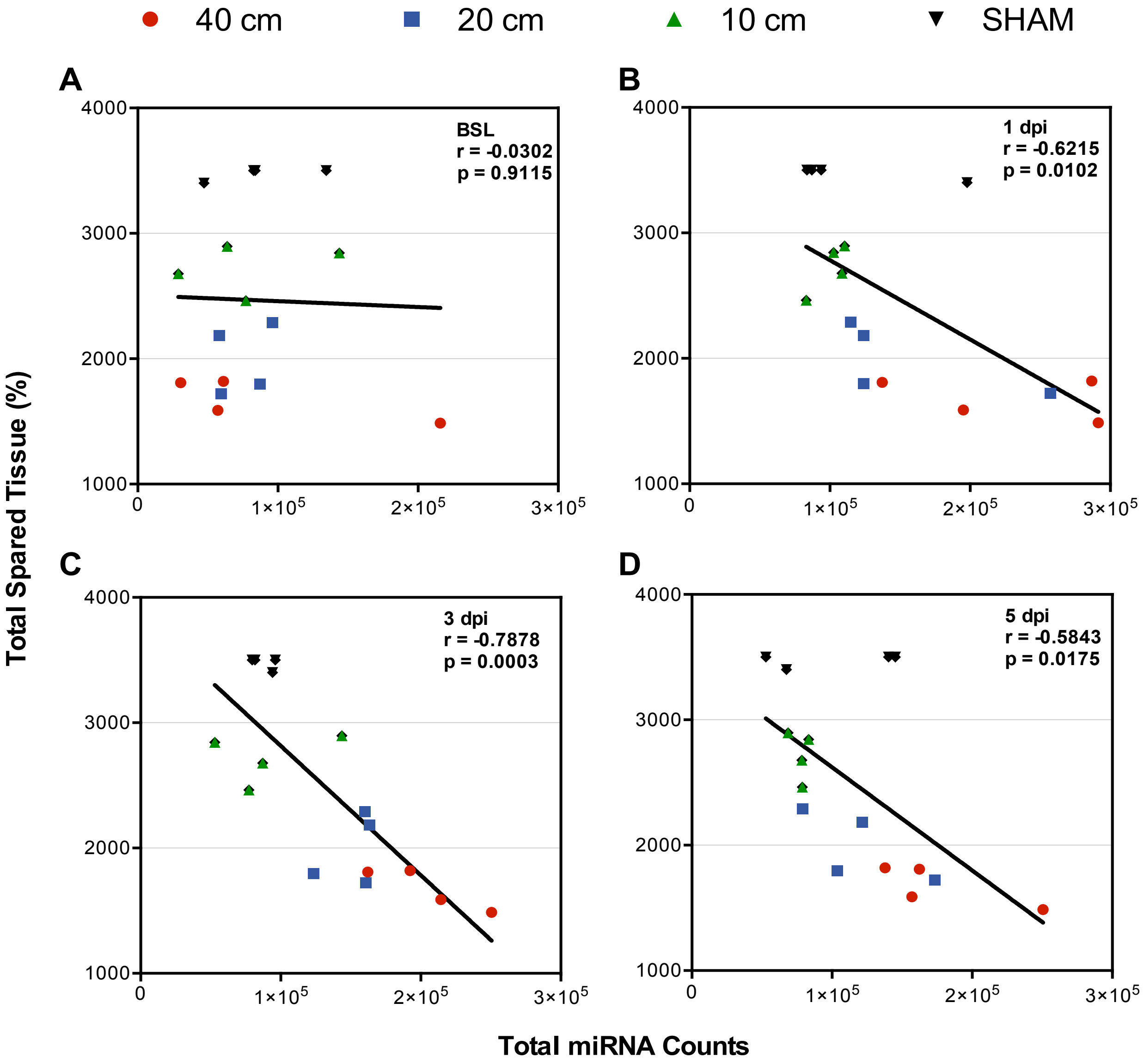

Supplementary Figure 6. Correlation between Force of Injury (N) and outcome parameters. Correlation between Force of injury and **A.** Porcine Thoracic Injury Behaviour Scores at 12 wpi and **B.** Total percent spared tissue. These figures simply demonstrate that for our pig model of thoracic SCI, the initial force of the contusion impact (dictated by the height of the weight drop) is strongly correlated to the degree of hindlimb locomotor impairment as measured by PTIBS **(A)** and the amount of tissue damage that occurs in the spinal cord **(B)**.

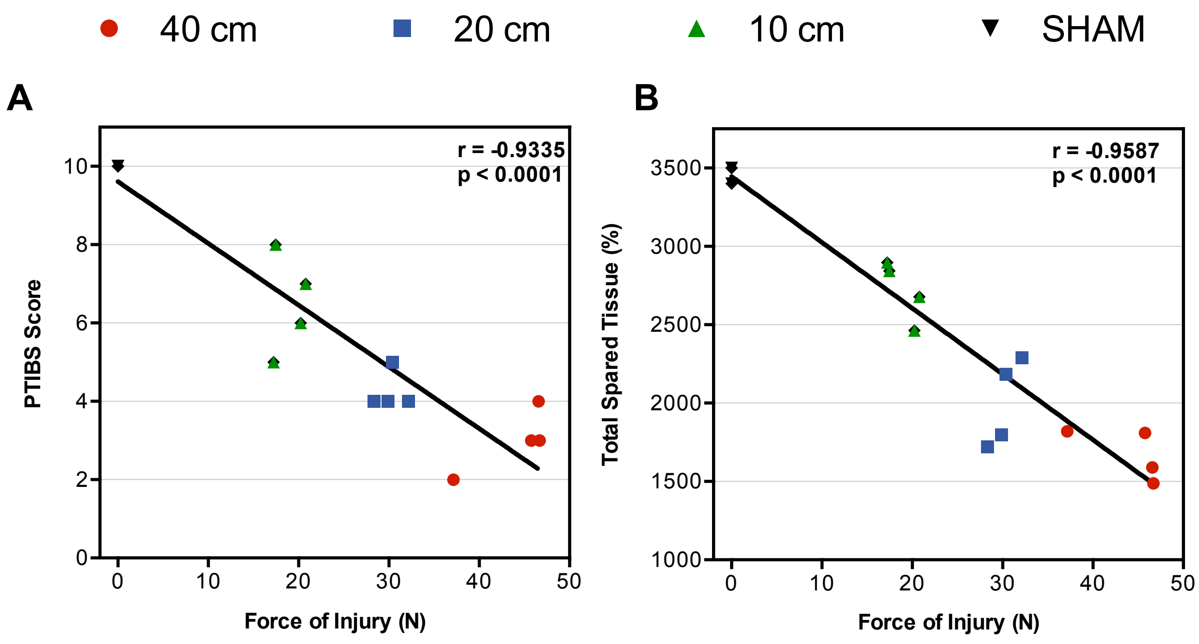

Supplement: Supplementary file 1 — Supplementary Figures & Tables [file 41598_2017_1299_MOESM1_ESM.pdf]
